# Effectiveness and safety of ultrasound-guided needle-knife therapy for patients with spinal pain disorders: a systematic review and meta-analysis

**DOI:** 10.3389/fmed.2025.1705669

**Published:** 2025-12-05

**Authors:** Do-Young Kim, Joon-Seok Lee, Sook-Hyun Lee, Yoon Jae Lee, Ju Yeon Kim, In Heo, Jae-Heung Cho, Byung-Kwan Seo, In-Hyuk Ha

**Affiliations:** 1 Department of Acupuncture and Moxibustion, Jaseng Korean Medicine Hospital, Seoul, Republic of Korea; 2 Department of Public Health Science, Seoul National University, Seoul, Republic of Korea; 3 Jaseng Spine and Joint Research Institute, Jaseng Medical Foundation, Seoul, Republic of Korea; 4 School of Korean Medicine, Pusan National University, Yangsan, Republic of Korea; 5 Department of Rehabilitation Medicine of Korean Medicine, Kyung Hee University, Seoul, Republic of Korea; 6 Department of Acupuncture and Moxibustion Medicine, College of Korean Medicine, Kyung Hee University, Seoul, Republic of Korea

**Keywords:** ultrasound-guided needle-knife therapy, spinal pain disorders, minimally invasive treatment, pain reduction, physical function improvement, systematic review and meta-analysis, adverse events

## Abstract

**Background:**

Requirements for the development of pre-surgical therapies for spinal pain disorders have arisen, with ultrasound-guided needle-knife therapy (US-NKT) gaining popularity as a minimally invasive treatment. This study aimed to evaluate the clinical outcomes of US-NKT, focusing on its effectiveness and safety.

**Methods:**

A comprehensive literature survey was conducted by using 12 databases to identify randomized controlled trials (RCTs) comparing the effects of US-NKT with control in patients with spinal pain disorders. Characteristics of RCTs were extracted. Also, a meta-analysis of pain and physical function (PF) outcomes was performed at 1-week, 1-month, and 3-month follow-ups. Adverse events (AEs) were also analyzed.

**Results:**

Of the 1,694 articles screened, 23 RCTs (*n* = 2,107) met the inclusion criteria, with 60.9% addressing spinal degenerative arthropathy. US-NKT significantly reduced pain at 1 week (standardized mean difference [SMD]: −1.11; 95% confidence interval [CI]: −1.42 to −0.79; I^2^ = 73%) and 1 month (SMD: −1.74; 95% CI: −2.50 to −0.98; I^2^ = 95%). However, the effects were not statistically significant at 3 months. PF improved significantly at all time .points, with the strongest effect at 1 week (SMD: −0.92; 95% CI: −1.42 to −0.42; I^2^ = 71%). US-NKT demonstrated superior benefits for pain and PF compared with recommended therapies (at all-time points) or conventional NKT (at 1 week and 1 month). AEs were reported in 43.5% of RCTs, with fewer incidents in the US-NKT groups (4.6%) compared with the controls (13.8%).

**Conclusion:**

US-NKT demonstrates superior efficacy in reducing pain and improving PF compared with recommended therapies or conventional NKT, with a favorable safety profile. However, sustained benefits beyond 3 months remain inconclusive.

**Systematic review registration:**

https://www.crd.york.ac.uk/prospero/, identifier CRD42024529315.

## 1 Introduction

Spinal pain disorders involve impairments in the vertebral complex, which maintains the body axis and facilitates the movement of the torso and limbs (1). According to the 2010 Global Burden of Disease Study, spinal pain accounted for 69.9% of all musculoskeletal disorders worldwide (2). With the increase in global life expectancy, the number of years lived with a disability due to back and neck pain has risen by approximately 50 and 75%, respectively, over the past decades since the 1990s (3, 4). In the United States, the societal burden of spinal pain, which significantly affects productivity, was estimated at $134 billion in 2016 (5, 6).

Surgical approaches are the primary treatment for spinal pain disorders, particularly in cases of severe neurological deficits, intolerable pain, or physical dysfunction resulting from structural defects of the spine (7). However, the preference for non-surgical treatments is driven by the vague long-term therapeutic superiority of surgery over conservative options, as well as concerns about the risks of complications and the potential failure to return to normal daily activities (8–10). Although epidural steroid injections (nerve blocks) are widely used as a pre-surgical treatment, their limitations, including side effects, significant variability in individual responses, and temporary effects, have been reported (11, 12). Consequently, interest in conservative treatments for spinal disorders is increasing, and surgical techniques are evolving to become less invasive and minimize postoperative complications (13).

Needle-knife therapy (NKT) is a minimally invasive surgical technique used for kinesiological rehabilitation that dissects and stimulates pathological tissues with a fine blade (14). It has been used clinically to treat musculoskeletal diseases, such as joint arthrosis, tendinopathy, spinal stenosis, and spondylosis (15). However, this procedure is associated with potential adverse events (AEs), including hematoma, nerve damage, and tissue rupture (16). To mitigate these risks, ultrasound-guided NKT (US-NKT), conducted under real-time imaging, has been introduced, making it more time- and cost-effective and safer than other imaging methods (17). Despite these advancements, evidence supporting the effectiveness and safety of US-NKT in treating spinal pain disorders remains limited.

In this review, we aimed to systematically evaluate the clinical outcomes of US-NKT, including its effectiveness and safety, in patients with spinal pain disease based on randomized controlled trials (RCTs). Additionally, we assessed the methodological applications of ultrasound in comparison with conventional NKT, focusing on its efficacy and physician strategies.

## 2 Materials and methods

This systematic review and meta-analysis was conducted in accordance with the Preferred Reporting Items for Systematic Reviews and Meta-Analyses guidelines (18). The protocol for this review was prospectively registered in PROSPERO.

### 2.1 Search strategy

A comprehensive literature search was performed using 12 electronic databases, including PubMed, Cochrane Library, Embase, Medline, Allied and Complementary Medicine Database, China National Knowledge Infrastructure, Scholarly and Academic Information Navigator, KoreaMed, KMbase, Oriental Medicine Advanced Searching Integrated System, ScienceON, and Korean Studies Information Service System, covering publications up to July 31, 2025. The major search terms included “spinal disease/disorder,” “spinal pain,” “needle knife,” “needle scalpel,” “ultrasound-guided,” and “randomized controlled trial.” The detailed search strategies are provided in Supplementary Table 1. The article type was restricted to RCTs, excluding gray literature, and studies in all languages were considered. Two authors (DY.K and SH.L) independently screened the titles and abstracts and identified RCTs that met the eligibility criteria.

### 2.2 Selection criteria

Eligibility screening was based on the following inclusion criteria: (1) published RCTs or randomized controlled crossover trials, (2) adult patients (≥ 18 years) with spine disorders, (3) studies employing US-NKT with flat-bladed surgical instrument as the intervention, and (4) a clinical question aimed at evaluating the therapeutic effect of the intervention compared with controls. The exclusion criteria were as follows: (1) articles without full text, (2) participants without spine-related diseases or in a postoperative condition, (3) studies lacking demographic information, (4) interventions not involving US-NKT or combined with other treatments (except for intervention-dependent aids such as local anesthesia), and (5) low-quality trials, defined as having a Physiotherapy Evidence Database (PEDro) scale score of < 5 points.

### 2.3 Quality assessment

The PEDro scale was used to assess the risk of bias and quality (19). The PEDro scale is a 10-point scale, in which properties such as randomization, allocation, similarity of baseline prognostic indicators, response rate, methodology for blinding, analysis, statistics, and measurement receive points. Consequently, trials with ≥ 5 points were considered high quality and were included for further data extraction.

### 2.4 Data extraction and analysis

Data on the participants’ demographic features, characteristics of spine disorders, intervention, control, measurement, and outcome data were then extracted, along with a statistical analysis of treatment effectiveness compared with the control. AEs were also recorded.

We judged the intervention efficacies as “Significant” or “Not significant” based on the data presentations of the original articles. In general, “Significant” meant that the intervention reached statistical significance (intervention vs. control, *P* < 0.05). Effective rate (ER) is defined as the proportion of patients who achieve a clinically meaningful improvement according to the original article.

The primary outcome of the meta-analysis was to estimate the overall treatment effect of US-NKT on pain and physical function (PF) compared with controls at 1 week, 1 month, and 3 months. Secondary outcomes included subgroup analyses comparing the treatment effect of US-NKT to the recommended treatment as per the Centers for Disease Control and Prevention guidelines in 2022 and NKT without US imaging guidance (20).

Meta-analyses were performed using Review Manager 5.4 software, following an intention-to-treat approach. The standardized mean difference (SMD) with a 95% confidence interval (CI) was used to express the effect sizes. Given the variability in the scales used across RCTs, continuous outcomes (pain and PF scores) were converted to a standardized 0–100 scale, where 0 indicated no symptoms and 100 represented the worst possible symptoms. The meta-analysis time points were selected based on the most frequently used intervals (1 week, 1 month, and 3 months). A random-effects model based on the DerSimonian and Laird method was used for all analyses (21). Given the expected clinical and methodological heterogeneity across the included studies, in terms of patient populations, intervention protocols, outcome measures, and comparator treatments, a random-effects model was employed. Statistical heterogeneity among studies was assessed using the I^2^ statistic with Review Manager 5.4 software, which quantifies the proportion of overall variation across studies due to heterogeneity rather than chance or random error. Funnel plot analysis for publication bias was practiced with the same software.

## 3 Results

### 3.1 Study identification and quality assessment

**FIGURE 1:**
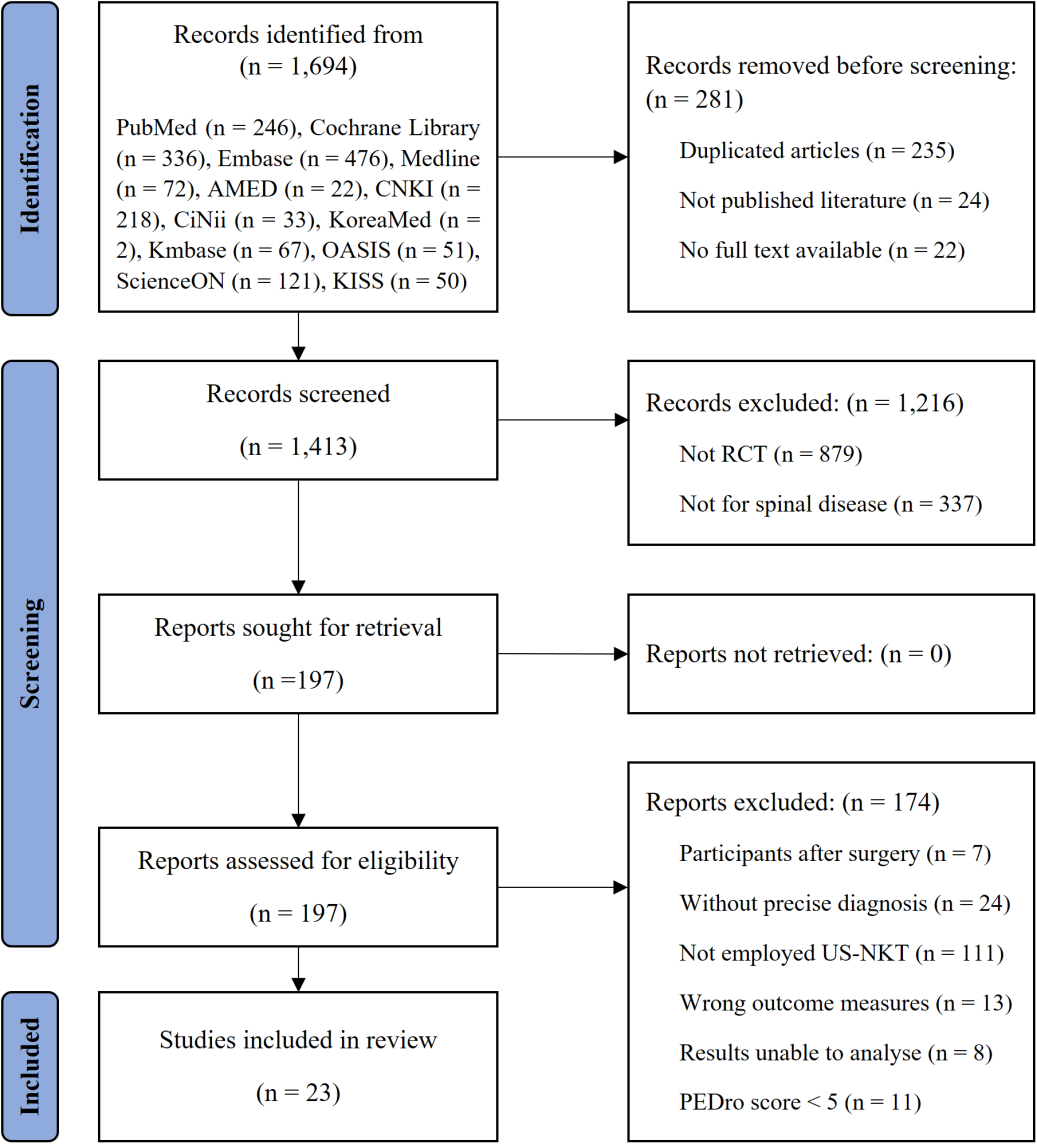
Flowchart of the study.

Of the 1,694 articles initially identified from the 12 electronic databases, 23 met the inclusion criteria after a thorough screening process (Figure 1). The mean PEDro score was 6.0 out of 10, with only three RCTs scoring above 6. The factor mainly influencing the scores was the blinding process. Due to the nature of the intervention, therapist blinding was impossible, while patient and assessor blinding played a critical role. The details of quality assessment for the studies are summarized in Supplementary Table 2.

### 3.2 Characteristics of participants

A total of 2,107 patients (mean age 45.4 ± 6.8 years) participated in the RCTs, presenting with cervical (16 RCTs, 69.6%), thoracic (one RCT, 4.3%), and lumbar spine complaints (seven RCTs, 30.4%). Arthropathy was the most common diagnosis, addressed in 14 RCTs (60.9%), followed by myopathy and spinal neuropathy (five RCTs each). Regarding the mean duration of the disease, sufferers with cervical spine disorders had a two-fold longer disease period than that of those with lumbar spine disorders (24.1 ± 16.3 *vs.* 13.0 ± 3.8 months).

### 3.3 Intervention and outcome measurements

The trend of disease duration extends to the number of interventions conducted and the treatment period (cervical: 2.7 ± 1.1 times, 2.4 ± 1.2 weeks/lumbar: 1.5 ± 0.9 times, 1.0 ± 1.6 weeks). Needle-knife procedures using a 1.0-mm diameter blade, the largest size reported, were used in only three RCTs (13.0%) for cervical disorders, whereas a 0.6 mm diameter was the most frequently used across studies (14 RCTs, 60.9%). In terms of outcome measurements, pain was the most commonly assessed parameter (20 RCTs, 87.0%), followed by ER in 14 RCTs (60.9%), PF (12 RCTs, 52.2%), disease-specific symptom scores (six RCTs, 26.1%), range of motion (ROM) (five RCTs, 21.7%), and hemodynamic parameters (three RCTs, 13.0%) (Table 1).

**TABLE 1:** Study characteristics.

Items	Count			
No. of RCT	23			
No. of participants (female)	2,107 (935^a^)			
Mean No. of participants (± SD)	91.6 ± 35.2			
Mean age (± SD)	45.4 ± 6.8^b^			
Target spine (No. of RCT, %)^c^	23 (100.0)			
Cervical spine	16 (69.6)			
Thoracic spine	1 (4.3)			
Lumbar spine	7 (30.4)			
Sacrum or coccyx	0 (0.0)			
**Type of disease^d^ (N. of RCTs, %)^c^**	**Cervical spine**	**Thoracic spine**	**Lumbar spine**	**Total**
Arthropathy	13	0	1	14 (60.9)
Myopathy	1	1	3	5 (21.7)
Neuropathy	2	0	3	5 (21.7)
Mean duration of disease (months ± SD)	24.1 ± 16.3	.	13.0 ± 3.8	21.1 ± 14.7
Mean N. of treatment time (± SD)	2.7 ± 1.1	3.0	1.5 ± 0.9	2.4 ± 1.2
Mean treatment period (weeks ± SD)	2.4 ± 1.2	3.0	1.0 ± 1.6	2.0 ± 1.4
**Diameter of needle (N. of RCTs, %)^e^**				
1.0 mm	3	0	0	3 (13.0)
0.6 mm	10	1	3	14 (60.9)
0.5 mm	1	0	0	1 (4.3)
Mean N. of measurements per RCT (± SD)	3.1 ± 0.8			
**Categories of measurement (N. of RCTs, %)^c^**				
Pain	20 (87.0)			
Effective rate (ER)	14 (60.9)			
Physical function (PF)	12 (52.2)			
Disease-specific symptom score	6 (26.1)			
Range of motion (ROM)	5 (21.7)			
Hemodynamics	3 (13.0)			

^a^This does not include three RCTs (37, 49, 61) of which sex information was not available.

^b^This is the mean of ages presented as median or mean in original articles.

^c^Some items have been applied multiple times in original articles, thus the total percentage is larger than 100%.

^d^This is categorized in 3 domains: arthropathy (spondylosis and osteoarthritis), myopathy (chronic neck pain, myofascial pain syndrome, and fasciitis) and neuropathy (herniated intervertebral disc and dorsal ramus syndrome).

^e^Some items have not been available in original articles, thus the total percentage is lesser than 100%.

### 3.4 Characteristics of RCTs

All RCTs included in this study were published since 2010, and 60.1% (14 RCTs) were published in the 2020s. The design and overall results of the RCTs are summarized in Table 2. Thirteen out of 16 RCTs for cervical disorders recruited participants diagnosed with spondylosis (22–34), whereas muscular pain disorders, such as fasciitis (35–37) and myofascial pain syndrome (38), were the major diseases diagnosed in RCTs with back spine complaints (four out of eight RCTs). The included RCTs primarily used NKT as a control intervention, whereas the remaining studies utilized standard recommended treatments such as acupuncture (23, 27, 29, 39), nerve blocks (28, 30–32, 40), laser therapy (32), and non-steroidal anti-inflammatory drugs (38).

**TABLE 2:** Summary of the RCTs with the intervention of US-NKT.

Disease (reference)	N. of participant (mean age)	Control (N.)	Diameter of needle (mm)/Frequency of US (MHz)/Treatment period (week)	Measurement (assessing time point, week)^a^	Statistical significance (applicable period, week)^b^
**Cervical spine**					
Spondylosis (22)	80 (55.0)	NKT (40)	0.6/6–13/4	Pain, PF, ROM (2, 4)	*P* < 0.05
Spondylosis (23)	106 (50.0)	Acupuncture (53)	0.6/7–12/3	Pain, ER, symptom score (1)	*P* < 0.05
Spondylosis (24)	100 (35.3)	NKT (50)	0.6/5–11/2	PF, ER, hemodynamics (1)	*P* < 0.05
Spondylosis (26)	200 (36.3)	NKT (100)	0.6/9–14/2	Pain, PF, EMG, lordosis angle (2, 12)	Pain, PF: *P* < 0.01; EMG, lordosis angle: *P* < 0.05
Spondylosis (25)	120 (46.7)	NKT (60)	0.5/6–13/2	ER, symptom score (0)	*P* < 0.05
Spondylosis (28)	82 (45.0)	Nerve block (41)	../../3	Pain, symptom score (1, 3)	*P* < 0.05
Spondylosis (27)	64 (34.2)	Acupuncture (32)	0.6/../4	Pain, PF, ER, symptom score (0)	*P* < 0.05
Spondylosis (29)	60 (57.4)	Acupuncture (30)	1.0/7–12/3	Pain, ER, symptom score (0)	Pain: *P* < 0.05;symptom score, ER: N.S
Spondylosis (31)	80 (49.1)	Nerve block (40)	0.6/../3	Pain, ER, ROM (1)	Pain, ROM: *P* < 0.05; ER: *P* < 0.01
Spondylosis (30)	58 (43.0)	Nerve block (29)	0.6/5–12/2	ER, symptom score, hemodynamics (0)	ER: N.S; symptom score, hemodynamics: *P* < 0.05
Spondylosis (32)	90 (43.0)	Laser (30); Nerve block (30)	0.6/../1 session	Pain, ROM, EMG (1)	Pain, ROM: *P* < 0.05; EMG: *P* < 0.01
HIVD (62)	100 (42.4)	NKT (50)	../../2	Pain, ER, ROM, N. Of tender point (0)	*P* < 0.05
Spondylosis (33)	80 (50.8)	NKT (40)	0.6/../1 session	Pain, ER, hemodynamics (0, 2, 4, 8)	Pain: *P* < 0.05 (0, 2, 4); ER: N.S; hemodynamics: *P* < 0.05 (8)
Spondylosis (34)	60 (48.2)	NKT (30)	1.0/6–13/3	Pain, ER (0, 1, 2, 4, 8)	*P* < 0.05
Chronic neck pain (39)	155 (40.6)	US-dry needling (73)	1.0/5–10/3	Pain, PF, mental health (12, 24)	Pain: *P* < 0.01; PF: *P* < 0.05; mental health: N.S
HIVD (49)	20 (..)	NKT (10)	0.6/7–14/1 session	Pain, PF (1)	N.S
**Thoracic spine**					
Myofascial pain syndrome (38)	100 (39.9)	Celecoxib (50)	0.6/../3	Pain, PF, mental health, inflammatory cytokine (3, 12)	*P* < 0.01
**Lumbar spine**					
Osteoarthritis (40)	118 (53.0)	Nerve block (59)	0.6/3–5/3	Pain, PF (0, 4)	Pain: *P* < 0.05 (4); PF: *P* < 0.05
Fasciitis (35)	78 (51.4)	NKT (39)	../5–12/1 session	Pain, PF, ER (1, 2, 4)	Pain: *P* < 0.01; PF, ER: *P* < 0.05
HIVD (48)	92 (46.9)	NKT (46)	0.6/6–13/4	Pain, PF, ER, ROM (0, 4, 12)	N.S
Fasciitis (36)	66 (51.1)	NKT (33)	../5–12/1 session	Pain, PF, ER, comfort (2, 4)	*P* < 0.05
Fasciitis (37)	60 (..)	NKT (30)	../../1 session	Pain, PF, ER (1, 2, 4)	Pain, ER: *P* < 0.05; PF: *P* < 0.05
Dorsal ramus syndrome (61)	118 (35.1)	X-ray-NKT (59)	../../1 session	Pain, ER (4, 12)	*P* < 0.05
HIVD (49)	20 (..)	NKT (10)	0.6/7–14/1 session	Pain, PF (1)	N.S

..: not available, EMG: electromyography, ER: effective rate, HIVD: herniated intervertebral disc, NKT: needle-knife therapy, N.S: not significant, PF: physical function, ROM: range of motion, US: ultrasound, US-NKT: ultrasound-guided needle-knife therapy. ^a^Period “0” indicates that the outcome assessment was conducted immediately after the treatment. ^b^Statistical significance applies to all measurements or periods unless otherwise noted in parentheses, such as for certain measurements or periods that were only applicable under mentioned conditions.

The overall results indicated that the measurements for pain, ER, and PF, the most frequently assessed domains, were statistically significant in 85.0, 71.4, and 75.0% of the relevant RCTs, respectively, for at least one measurement period. Notably, three of the four RCTs involving patients with herniated intervertebral discs (HIVDs) showed no statistically significant therapeutic effect of US-NKT in any measurement domain (Table 2).

### 3.5 Effect of US-NKT on pain and PF

**FIGURE 2:**
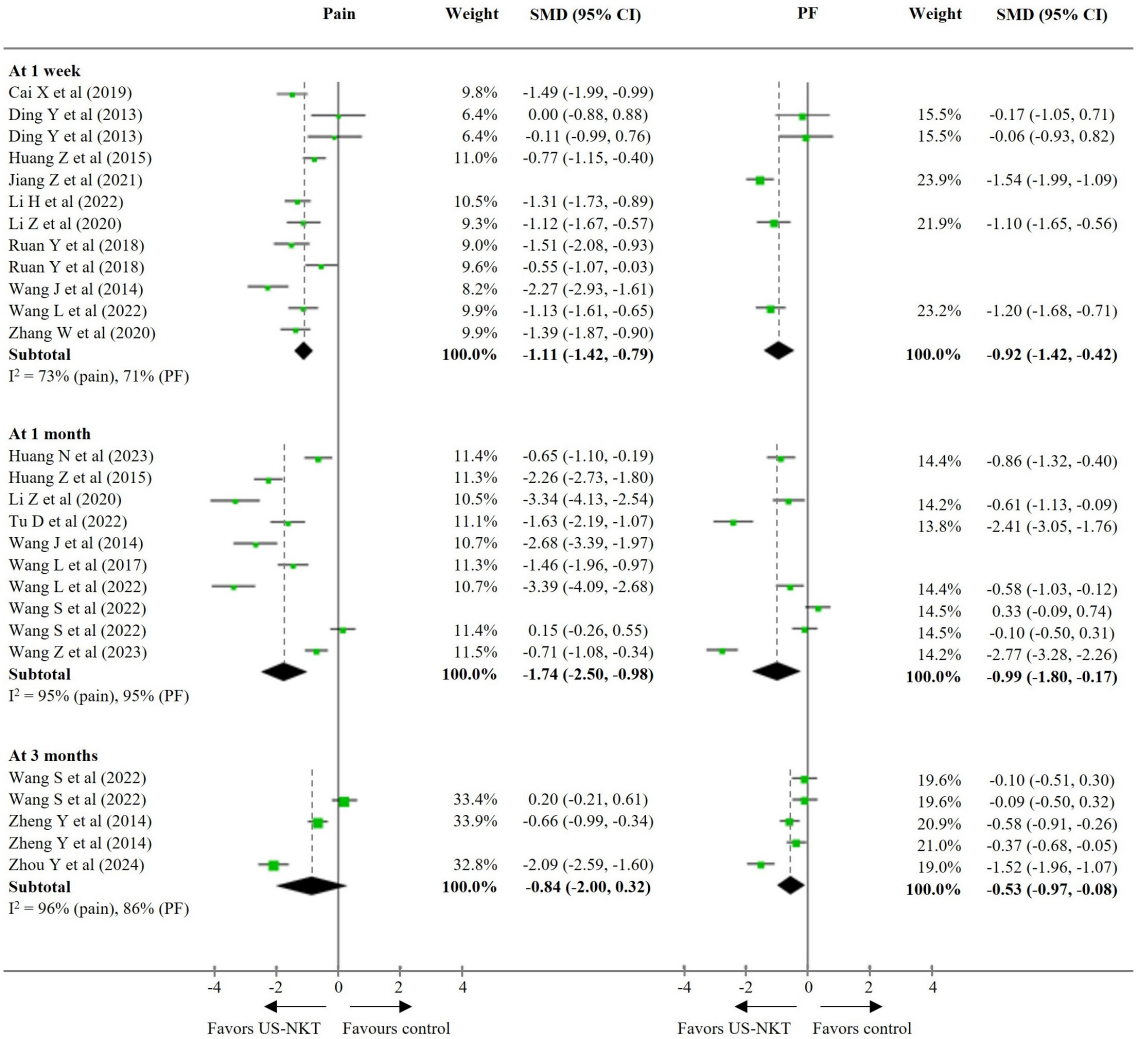
SMD for improvement in pain and function for US-NKT versus control for spine disorders. CI, confidence interval; PF, physical function; SMD, standardized mean difference; US-NKT, Ultrasound-guided needle knife therapy.

Seventeen RCTs were included in the primary meta-analysis that compared US-NKT to all controls, with pooled results for pain and PF standardized on a 0–100 scale. In terms of pain reduction, US-NKT demonstrated a superior effect at 1 week, with a SMD of −1.11 points (95% CI: −1.42 to −0.79; I^2^ = 73%) compared with the control group. This difference was more pronounced at the 1-month follow-up (SMD −1.74; 95% CI: −2.50 to −0.98; I^2^ = 95%), while the difference at 3 months was not statistically significant (SMD −0.84; 95% CI: −2.00 to 0.32; I^2^ = 96%). Regarding PF, the effect size over time followed a pattern similar to that observed for pain, with statistically significant results at 1 week (SMD −0.92; 95% CI: −1.42 to −0.42; I^2^ = 71%), 1 month (SMD −0.99; 95% CI: −1.80 to −0.17; I^2^ = 95%), and 3 months (SMD −0.53; 95% CI: −0.97 to −0.08; I^2^ = 86%) (Figure 2).

**FIGURE 3:**
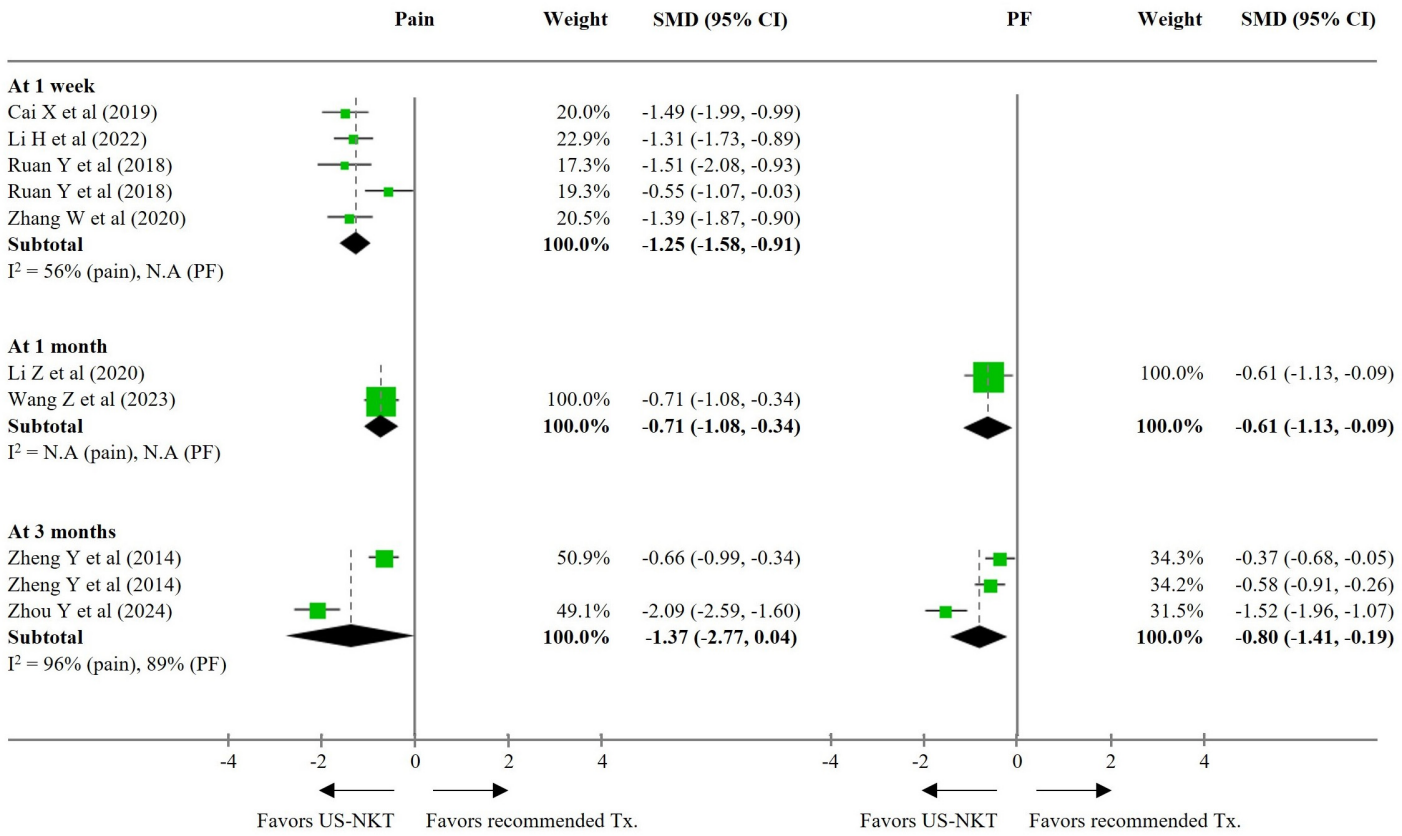
SMD for improvement in pain and function for US-NKT versus recommended treatment for spine disorders. CI, confidence interval; N.A, not available; PF, physical function; SMD, standardized mean difference; Tx., treatment; US-NKT, Ultrasound-guided needle knife therapy.

**FIGURE 4:**
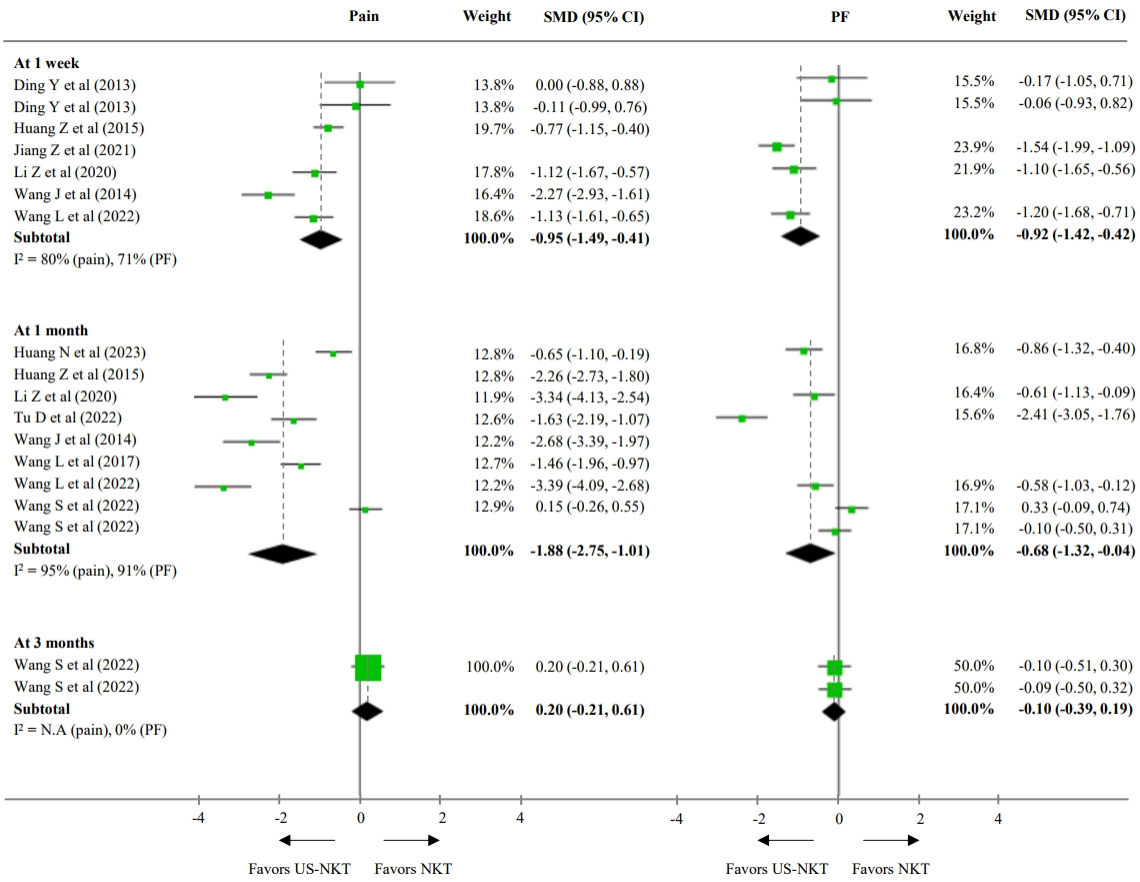
SMD for improvement in pain and function for US-NKT versus NKT for spine disorders. CI, confidence interval; N.A, not available; PF, physical function; NKT, needle knife therapy; SMD, standardized mean difference; US-NKT, Ultrasound-guided needle knife therapy.

For the secondary outcome analysis, comparisons were made with results from eight RCTs using the recommended treatments and 10 trials using conventional NKT as controls (Figures 3, 4). Compared with established therapies, US-NKT demonstrated a greater effect on pain reduction at 1 week, with an SMD of −1.25 points (95% CI: −1.58 to −0.91; I^2^ = 56%). This finding is further supported by limited evidence for both pain and PF at the 1− and 3-month follow-ups (at 3 months, pain: SMD −1.37; 95% CI: −2.77 to 0.04; I^2^ = 96%/PF: SMD −0.80; 95% CI: −1.41 to −0.19; I^2^ = 89%) (Figure 3). Regarding the clinical advantage of US guidance, statistically significant improvements were observed in both pain and PF at 1 week (pain: SMD −0.95; 95% CI: −1.49 to −0.41; I^2^ = 80%/PF: SMD −0.92; 95% CI: −1.42 to −0.42; I^2^ = 71%) and at 1 month (pain: SMD −1.88; 95% CI: −2.75 to −1.01; I^2^ = 95%/PF: SMD −0.68; 95% CI: −1.32 to -0.04; I^2^ = 91%). However, these effects were not statistically significant at 3 months (Figure 4).

### 3.6 AEs reported in RCTs

Ten of 23 RCTs (43.5%) reported AEs. Among the six studies that conducted a statistical analysis of AEs, four reported that the US-NKT group had fewer AEs than the control group. The incidence of AEs was approximately three times higher in the control group than in the intervention group (4.6% in the US-NKT group vs. 13.8% in the control group), with most AEs being localized reactions such as pain and bruising. Although unintended neurological AE, such as paralysis in the limbs and nerve injuries, occurred only in the control group, serious AEs were not reported in either group (Table 3).

**TABLE 3:** Summary of cases of AEs in the RCTs.

Disease (reference)	N. of participants/ Control (N.)	Diameter of needle (mm)	Summary of description for AE in RCTs^a^			Additional intervention
			Intervention group N. of case (%), event	Control group N. of case (%), event	Statistical significance	
**Cervical spine**						
Chronic neck pain (39)	155/US-dry needling (73)	1.0	6 (7.3%), local pain or somatic reactions	7 (9.6%), local pain or somatic reactions	..	None
Spondylosis (25)	120/NKT (60)	0.5	1 (1.8%), bruise	6 (11.3%), bruise	*P* < 0.05	None
Spondylosis (27)	64/acupuncture (32)	0.6	4 (12.5%), bruise; 1 (3.1%), local pain	3 (9.4%), bruise; 3 (9.4%), headache	N.S	None
Spondylosis (30)	58/nerve block (29)	0.6	2 (6.9%), discomfort; 1 (3.4%), local pain; 1 (3.4%), bruise	6 (20.7%), discomfort; 5 (17.2%), local pain; 4 (13.8%), bruise	*P* < 0.05	None
Spondylosis (34)	60/NKT (30)	1.0	None	3 (10.0%), local edema	..	Local anesthesia^b^
**Thoracic spine**						
Myofascial pain syndrome (38)	100/celecoxib (50)	0.6	Some patients (..), bruise	5 (10.0%), gastrointestinal reactions	..	Local anesthesia^b^
**Lumbar spine**						
Osteoarthritis (40)	118/nerve block (59)	0.6	1 (1.7%), muscle spasm; 1 (1.7%), subcutaneous hemorrhage	1 (1.7%), palpitation; 1 (1.7%), rash	.	Local anesthesia^b^
Fasciitis (35)	78/NKT (39)	..	1 (2.6%), headache	3 (7.7%), headache; 3 (7.7%), paralysis in limb; 2 (5.1%), others	*P* < 0.05	None
HIVD (48)	92/NKT (46)	0.6	None	2 (4.4%), nerve injury	N. S	Local anesthesia^b^
Fasciitis (37)	60/NKT (30)	..	2 (6.7%), dizziness and vomit	4 (13.3%), local pain; 2 (6.7%), headache and vomit; 1 (3.3%), paralysis in limb; 1 (3.3%), other	*P* < 0.05	Local anesthesia^b^

..: not available; AE, adverse event; HIVD, herniated intervertebral disc; NKT, needle-knife therapy; N.S, not significant; US, ultrasound. ^a^The data under this item category are derived from the original article. ^b^This had been used as supportive medication for the intervention practice.

## 4 Discussion

All the included RCTs targeted musculoskeletal diseases, especially those caused by degeneration within the spinal complex, which is an indication of NKT (15). The prevalence of spinal degeneration due to aging, overuse, or obesity has been increasing, affecting approximately one-third of individuals aged ≥ 65 years in the United States (41, 42). With regard to the treatment of spinal pain, the gap between conservative treatment and surgery has emerged as a growing medical issue, emphasizing cost-effectiveness and safety (43). In this study, we analyzed the therapeutic effects of US-NKT and the overall characteristics of RCTs conducted to date on patients with spinal pain disorders.

The underlying mechanisms of degenerative spinal pain include discogenic inflammation, nerve compression, and sprain of the soft tissues (44). This pain is frequently accompanied by movement restrictions, which lead to a decline in daily tasks and social activities (45). Our results demonstrated a significant therapeutic effect of US-NKT on both pain and PF at 1 week and 1 month, with the effect on PF extending to 3 months. By making an incision and promoting the restoration of adhesive lesions, NKT has been frequently employed for musculoskeletal rehabilitation of PF (46). This might explain why US-NKT, and not conventional NKT, as in our data, showed better effects at the 3-month follow-up than the recommended therapy. This is further supported by RCT results that assessed ROM or spinal alignment angle, with 83.3% of the studies (five out of six RCTs) reporting a statistically significant improvement (Table 2).

Arthropathy and myopathy were the primary conditions targeted by the RCTs for the cervical and lumbar spine, respectively (Table 1). Given the more intensive interventions required to treat degenerative arthropathy, this may explain the longer disease duration observed in the cervical spine, resulting in an almost two-fold increase in the treatment duration (47). Significant clinical improvements were observed at 1 week and 1 month (except for PF in the cervical spine at 1 week), extending to 3 months only in the cervical spine (Supplementary Figures 1, 2). This trend was influenced by all three RCTs for patients with HIVD included in the meta-analysis, which reported no statistically significant differences in pain and PF outcomes compared with conventional NKT (48, 49). In terms of US-guided intervention strategies, while this approach allows physicians to locate target lesions and avoid nearby vulnerable structures (Supplementary Table 3), US-NKT does not present a clear advantage over conventional methods in patients with HIVDs. This is largely because it cannot directly access the spinal canal where the herniated disc is located and hardly has a faster therapeutic effect than surgeries (50). Also, this suggests that NKT may play a more efficacious role in anti-nociceptive pain associated with degenerative arthropathy or myofascial pain syndrome than in neuropathic pain.

We conducted a review of US-guided strategies employed by RCTs (Supplementary Table 3). Except for one RCT (40) that used a relatively low frequency of 3–5 MHz to target the lumbar spine articular capsule, all other RCTs used frequencies of 5–14 MHz, which is a range traditionally applied for musculoskeletal disorders (51). Myopathies and degenerative arthropathy can be identified on ultrasonography by heterogeneous echogenicity of trigger points, contracture knots, or adhesive lesions, and by features such as capsular thickening, irregular joint margins, and joint space narrowing, respectively (52, 53). Given that eliciting a local twitch response (LTR) in myopathies is critical for pain relief and functional recovery, US-guided lesion identification significantly enhances therapeutic outcomes (54). According to pain modulation theories, the identification of both trigger points and pathological facet joints, sources of synergistic spinal referral pain, is essential for managing chronic back pain and neuropathic syndromes (55, 56).

Of the 23 RCTs in this review, 14 identified target lesions via ultrasonography: muscle fibers with nodules or trigger points (7 RCTs), spine articular capsules (2 RCTs), and nerve tissues (2 RCTs) (Supplementary Table 3). RCTs targeting muscle fibers or capsules showed statistically significant improvements in pain and ER compared to controls (26–28, 32, 34, 37–40). In contrast, those targeting nerve tissues such as nerve root or stellate ganglion failed to present benefit on general improvement (30, 33). These findings suggest that NKT is more effective for releasing contracted soft tissues than for neuromodulation. Similarly, US-guided dry needling (DN) may achieve comparable effects by inducing LTR, though NKT may offer superior outcomes due to its use of flat-bladed and larger-diameter needles which is effective for tissue dissection (57, 58).

Several studies have also used US for prognostic assessment, such as monitoring the alleviation of pathological lesions following treatment (24, 38) or evaluating hemodynamics using Doppler tests, which demonstrated significant improvements in blood flow related to the cervical spine (24, 30, 33) (Supplementary Table 3). Contrary to our expectations, US-guided NKT did not show significant benefits in reducing AEs related to neurovascular stimulation, such as headaches or somatic reactions. However, approximately one-third of the peripheral AEs, including subcutaneous hemorrhage and local pain, which are mainly regarded as AE of NKT, were reported less frequently in the US-NKT groups than in the controls, suggesting its advantage in minimizing damage to unintended tissues (59) (Table 3).

Our findings suggest that US-NKT is more effective in reducing pain and improving PF and symptom scores than nerve block therapy, an established pre-surgical treatment for spinal pain disorders, based on three RCTs (Table 2). In the context of chronic spinal pain disorders, safety concerns surrounding the long-term use of steroidal drugs, particularly in older adults or patients with diabetes, as well as the potential for delaying surgery, are increasingly recognized (60). In this regard, US-NKT could provide patients with degenerative spinal disorders with a cost-effective and safe therapeutic option as a minimally invasive surgical procedure with a reduced learning curve for physicians.

Our study had some limitations. First, this review highlights the paucity of high-quality RCTs despite applying a cutoff score of 5 on the PEDro scale. The mean PEDro score across the included studies was 6.0, which approaches the minimum threshold for good quality and is considered to be at the lower border of this category (Supplementary Table 2). This may be attributed to the fact that most studies have been published within the last decade, reflecting the recent emergence of this treatment method and consequently resulting in a limited body of literature. Second, considerable heterogeneity was observed in the available data. To address this, subgroup analyses were performed based on spinal regions and types of control treatments; however, significant heterogeneity persisted despite these efforts. This variability arose from the absence of a distinction between different disease types, variations in PF scales, and types of control treatments during the data pooling process. These factors may also influence the interpretation of our funnel plot analysis, which showed asymmetry suggestive of potential publication bias (Supplementary Figure 3). Third, comparative analyses of patient-important outcomes such as return to work, surgery avoidance, and clinically relevant procedural endpoints (e.g., LTR and palpable release) were limited. These outcomes could offer valuable practical insights for clinicians and researchers regarding the clinical applicability of US-NKT. Fourth, no data beyond 3 months were available, limiting assessment of the durability of treatment effects. Lastly, only 43.5% of the included RCTs reported AEs, and none employed standardized AE classification systems. This suggests a potential underreporting bias, which may limit the reliability of the safety profile.

To our knowledge, this is the first study to evaluate the efficacy of US-NKT for spinal pain disorders across global RCTs. It provides a foundational basis for future research targeting specific populations, disease entities, instrument parameters, lesion sites, and functional restoration goals. Further clinical studies are warranted to establish standardized intervention protocols, including needle specifications, ultrasound frequency, imaging guidance, and treatment sessions. In particular, to enhance reproducibility and clinical applicability, future investigations should incorporate functional ultrasound illustrations to visualize dynamic muscle and fascial movements, currently a limitation in existing studies, and standardize procedural endpoints such as LTR and palpable release. Additionally, well-designed trials comparing US-NKT with established evidence-based therapies, incorporating long-term follow-up and adherence to international safety monitoring standards, are needed to enable a balanced evaluation for clinical application.

## 5 Conclusion

This systematic review and meta-analysis integrates findings from previous RCTs investigating benefits of US-NKT for spinal pain disorders. The results indicate that US-NKT significantly reduces pain and physical disability at both 1 week and 1 month compared to controls, including recommended therapies. Furthermore, US-NKT demonstrates a favorable safety profile with fewer adverse events than controls. However, the long-term efficacy of US-NKT beyond 3 months remains inconclusive. Future research is needed to explore disease-specific, and target tissue-specific treatment approaches and mechanisms.

## Data Availability

The raw data supporting the conclusions of this article will be made available by the authors, without undue reservation.
