# Supplementary material for: Effectiveness and safety of ultrasound-guided needle-knife therapy for patients with spinal pain disorders: a systematic review and meta-analysis
Source: Front Med (Lausanne). 2025 Dec 5;12:1705669. doi: 10.3389/fmed.2025.1705669 (PMC12714934; doi:10.3389/fmed.2025.1705669)
Supplement: Supplementary file 1 [file Data_Sheet_1.docx]

**Supplementary Fig. 1. SMD for improvement in pain and function for US-NKT versus control for cervical spine disorders**

CI, confidence interval; N.A, not available; PF, physical function; SMD, standardized mean difference; US-NKT: Ultrasound-guided needle knife therapy


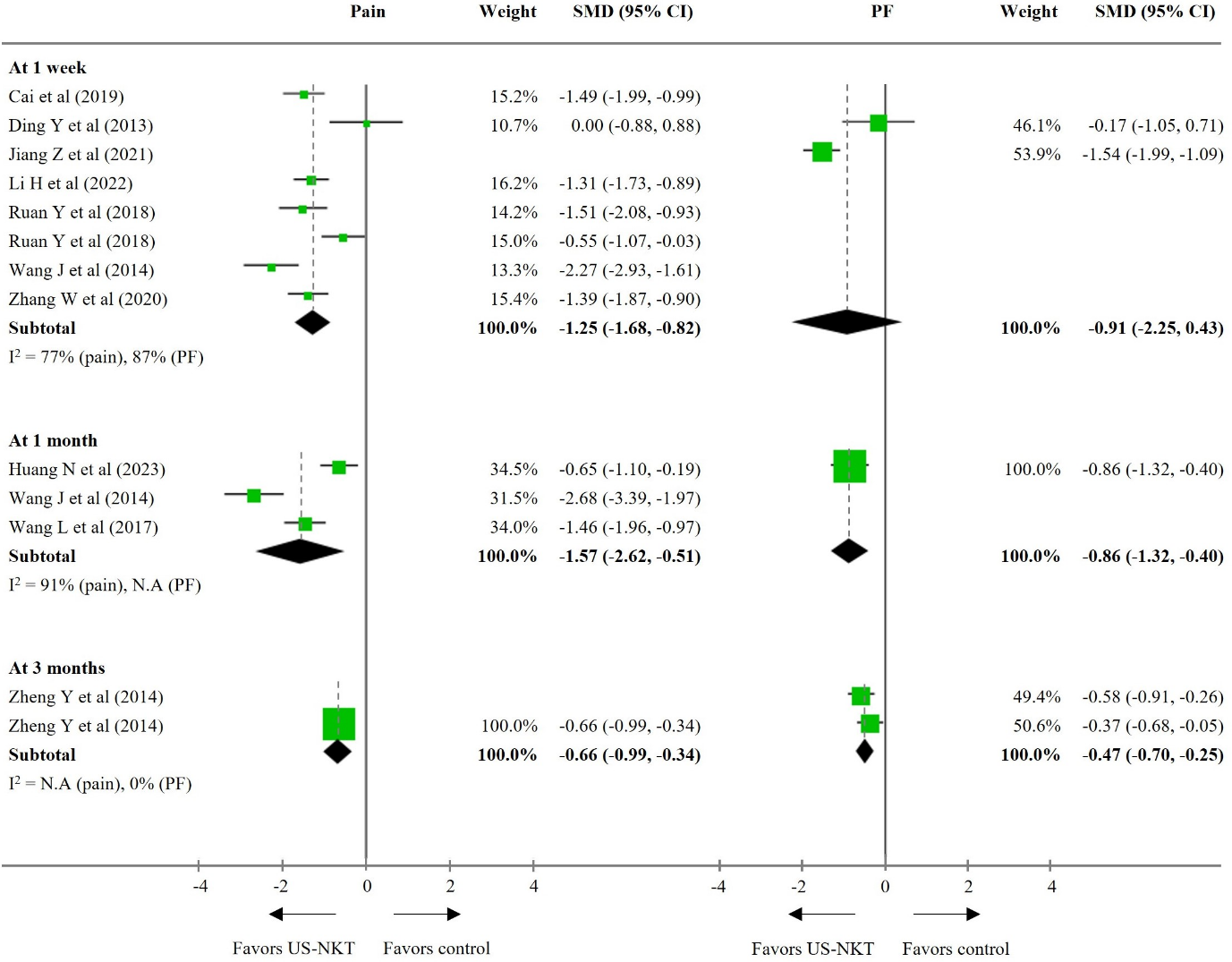


**Supplementary Fig. 2. SMD for improvement in pain and function for US-NKT versus control for thoracic and lumbar spine disorders**

CI, confidence interval; PF, physical function; SMD, standardized mean difference; US-NKT: Ultrasound-guided needle knife therapy


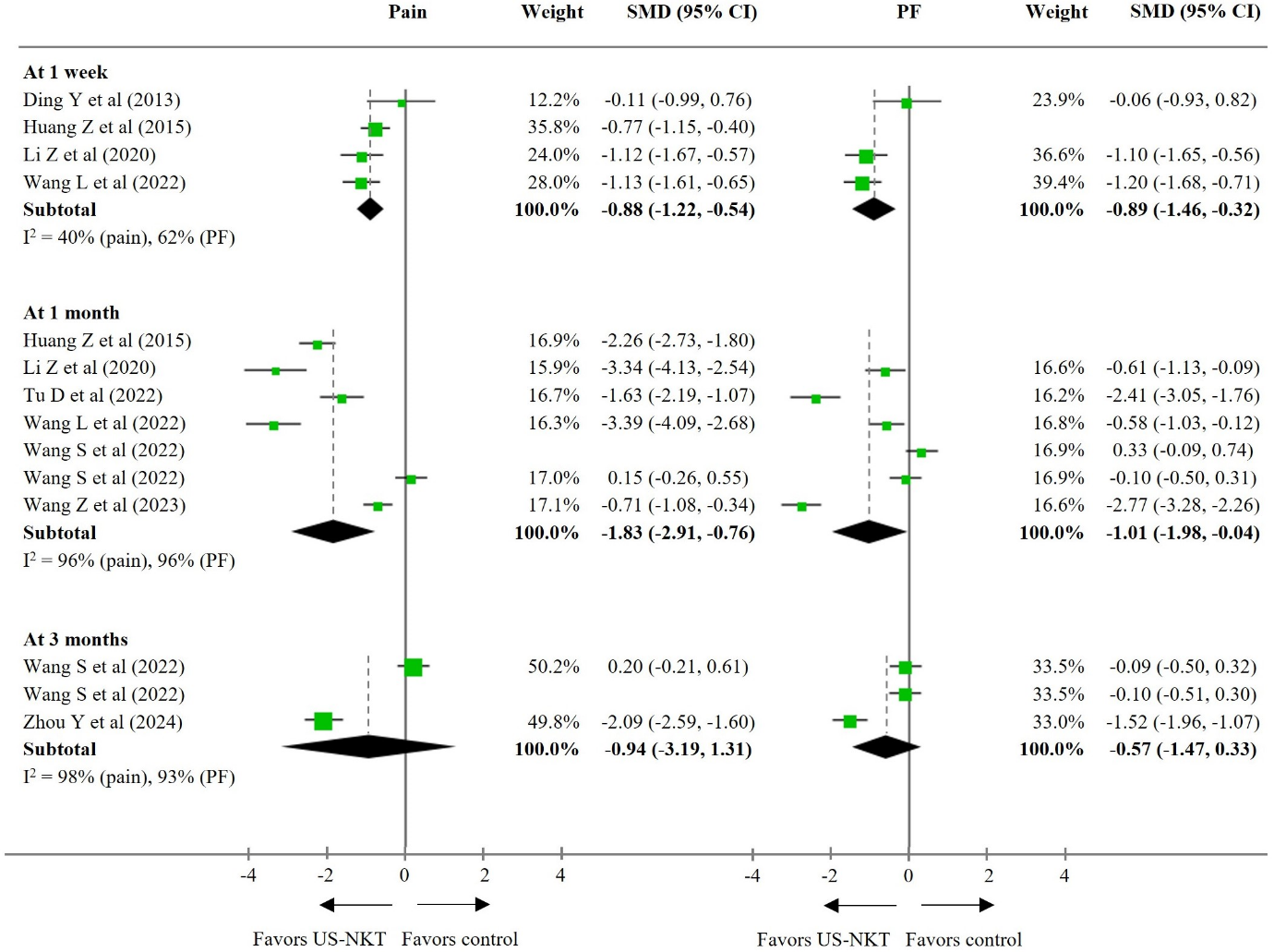


**Supplementary Fig. 3. Funnel plot for improvement in pain and function for US-NKT versus control for spine disorders**

Pain (left), Physical function (right).


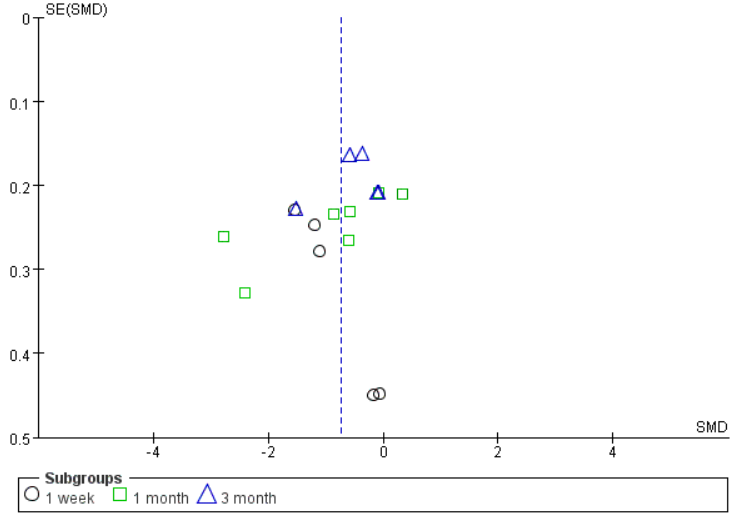

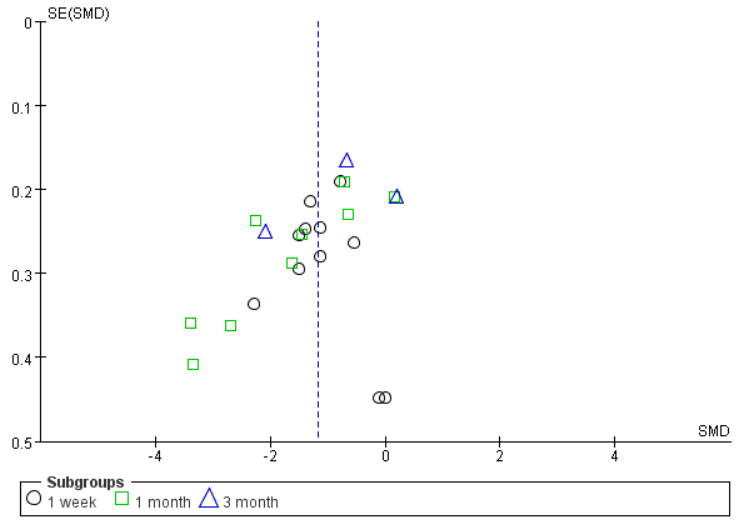


**Supplementary Table 1. Searching strategy for literature survey**

| Search terms |
| --- |
| Participants (or)  “cervical vertebrae,” “thoracic vertebrae,” “lumbar vertebrae,” “axial,” “cervical,” “thoracic,” “lumbar,” “sacrum,” “sacral,” “coccyx,” “coccygeal,” “vertebra,” “vertebrae,” “vertebral,” “intervertebra,” “radiculopathy,” “radiculitis,” “radicular pain,” “myofascial pain syndrome,” “spondylosis,” “spondylitis,” “spondylolisthesis,” “degeneration,” “degenerative,” “stenosis,” “bulging,” “bulged,” “bulge,” “disc,” “discs,” “disk,” “disks,” “herniation,” “spinal pain,” “spinal disorder,” “spinal disease,” “spinal disability,” “neck pan,” “cervical pain,” and “back pain.”  Intervention (or)  “needle knife,” “acupotome,” “acupotomy,” “cutting needle,” “needle scalpel,” “acupotomlogy,” “miniscalpel,” “sword like needle,” “acupuncture,” “needling,” “acupuncture point,” “ultrasound,” “ultrasound-guided,” and “Ultrasonography.”  Study design (or)  “randomized controlled trial,” “clinical trial,” “randomized,” “randomly,” and “trial.” |
| Participants (and) Intervention (and) Study design |

**Supplementary Table 2. Risk of bias and study quality of RCTs on the PEDro scale**

| Author (year) | Random allocation | Concealed allocation | Baseline similarity | Subject blinding | Therapist blinding | Assessor blinding | <15% dropouts | Intention to treat analysis | Between-group difference reported | Point estimate, variability reported | Total  (0–10)^a^ |
| --- | --- | --- | --- | --- | --- | --- | --- | --- | --- | --- | --- |
| Zhou Y (2024) | Y | Y | Y | Y | N | Y | Y | Y | Y | Y | 9 |
| Huang N (2023) | Y | N | Y | N | N | N | Y | N | Y | Y | 5 |
| Wang Z (2023) | Y | Y | Y | N | N | N | Y | N | Y | Y | 6 |
| Wang L (2022) | Y | N | Y | N | N | N | Y | N | Y | Y | 5 |
| Wang S (2022) | Y | N | Y | N | N | N | Y | N | Y | Y | 5 |
| Li H (2022) | Y | Y | Y | N | N | N | Y | N | Y | Y | 6 |
| Tu D (2022) | Y | Y | Y | N | N | N | Y | N | Y | Y | 6 |
| Jiang Z (2021) | Y | Y | Y | N | N | N | Y | N | Y | Y | 6 |
| Liu H (2021) | Y | Y | Y | N | N | N | Y | N | Y | Y | 6 |
| Gao X (2021) | Y | Y | Y | N | N | N | Y | N | Y | Y | 6 |
| Zhang W (2020) | Y | Y | Y | N | N | N | Y | N | Y | Y | 6 |
| Jiang Z (2020) | Y | Y | Y | N | N | N | Y | N | Y | Y | 6 |
| Li Z (2020) | Y | Y | Y | N | N | N | Y | N | Y | Y | 6 |
| Zhang S (2020) | Y | N | Y | N | N | N | Y | N | Y | Y | 5 |
| Cai X (2019) | Y | N | Y | N | N | N | Y | N | Y | Y | 5 |
| Zhong Z (2019) | Y | Y | Y | N | N | N | Y | N | Y | Y | 6 |
| Ruan Y (2018) | Y | Y | Y | Y | N | Y | Y | N | Y | Y | 8 |
| Liu H (2018) | Y | Y | Y | N | N | N | Y | N | Y | Y | 6 |
| Wang L (2017) | Y | Y | Y | N | N | N | Y | N | Y | Y | 6 |
| Huang Z (2015) | Y | N | Y | N | N | N | Y | N | Y | Y | 5 |
| Zheng Y (2014) | Y | Y | Y | Y | N | Y | Y | N | Y | Y | 8 |
| Wang J (2014) | Y | Y | Y | N | N | N | Y | N | Y | Y | 6 |
| Ding Y (2013) | Y | Y | Y | N | N | N | Y | N | Y | Y | 6 |

^a^Item 1 (specification of eligibility of criteria), which relates to external validity, is not counted in the overall score, which ranges from 0 to 10.

**Supplementary Table 3. Strategy for US guidance in RCTs**

| Author (year) | Disease (type of spine) | Frequency of ultrasound | Target lesion with identification^a^ | Prognostic assessment |
| --- | --- | --- | --- | --- |
| Zhou (2024) | Myofascial pain syndrome (T) | ·· | Trigger point | Alleviation of myofascial tension |
| Huang N (2023) | Spondylosis (C) | 6–13 MHz | ·· | ·· |
| Wang Z (2023) | Osteoarthritis (L) | 3–5 MHz | Spine articular capsule | ·· |
| Wang L (2022) | Fasciitis (L) | 5–12 MHz | ·· | ·· |
| Wang S (2022) | HIVD (L) | 6–13 MHz | Lamina and ligamentum flavum | ·· |
| Li H (2022) | Spondylosis (C) | 7–12 MHz | ·· | ·· |
| Tu D (2022) | Fasciitis (L) | 5–12 MHz | ·· | ·· |
| Jiang Z (2021) | Spondylosis (C) | 5–11 MHz | ·· | Pathologic lesion imaging, hemodynamics |
| Liu H (2021) | Spondylosis (C) | 9–14 MHz | Adhesion, proliferation, and hypertrophy of muscle fiber | ·· |
| Gao X (2021) | Spondylosis (C) | 6–13 MHz | Stenosis lesion | ·· |
| Zhang W (2020) | Spondylosis (C) | ·· | Origins and insertions of paraspinal muscles | ·· |
| Jiang Z (2020) | Spondylosis (C) | ·· | Adhesion or nodule of muscle fiber | ·· |
| Li Z (2020) | Fasciitis (L) | ·· | Nodule of muscle fiber | ·· |
| Zhang S (2020) | Spondylosis (C) | 7–12 MHz | ·· | ·· |
| Cai X (2019) | Spondylosis (C) | ·· | Soft tissues around the transverse process | ·· |
| Zhong Z (2019) | Spondylosis (C) | 5–12 MHz | Stellate ganglion | Hemodynamics |
| Ruan Y (2018) | Spondylosis (C) | ·· | Nodules around the joint | ·· |
| Liu H (2018) | HIVD (C) | ·· | ·· | ·· |
| Wang L (2017) | Spondylosis (C) | ·· | Nerve root | Hemodynamics |
| Huang Z (2015) | Dorsal ramus syndrome (L) | ·· | ·· | ·· |
| Zheng Y (2014) | Chronic neck pain (C) | 5–10 MHz | Trigger point | ·· |
| Wang J (2014) | Spondylosis (C) | 6–13 MHz | Capsule of facet joint | ·· |
| Ding Y (2013) | HIVD (C, L) | 7–14 MHz | ·· | ·· |

··: not available, C: cervical spine, HIVD: herniated intervertebral disc, L: lumbar spine, T: thoracic spine, US: ultrasound.

^a^The data under this item are available information derived from the original article.
